# Survival and quality-adjusted life years in critically ill cancer patients: analysis on the fifth day of intensive care unit stay

**DOI:** 10.62675/2965-2774.20260443

**Published:** 2026-08-07

**Authors:** Miriam Cristine Vahl Machado, Antônio Carlos Pedroso-de-Lima, Gisela Tunes da Silva, Alexandre Biasi Cavalcanti

**Affiliations:** 1 Intensive Care Unit Centro Hospitalar Unimed Joinville SC Brazil Intensive Care Unit, Centro Hospitalar Unimed - Joinville (SC), Brazil.; 2 Universidade de Sao Paulo Institute of Mathematics and Statistics Department of Statistics São Paulo SP Brazil Institute of Mathematics and Statistics, Department of Statistics, Universidade de Sao Paulo - São Paulo (SP), Brazil.; 3 Research Institute HCor-Hospital do Coração São Paulo SP Brazil Research Institute, HCor-Hospital do Coração - São Paulo (SP), Brazil.

## INTRODUCTION

Assessing the prognosis of critically ill cancer patients is challenging due to the heterogeneity of the oncologic conditions and the influence of acute organ dysfunction on outcomes.^(1)^ Beyond survival, health-related quality of life (HRQoL) provides patient-centered insights into post-critical illness trajectories, guiding clinician and patient decisions.^(2-4)^ By combining survival time and HRQoL, we can estimate the Quality-Adjusted Life Years (QALY), in which one year of survival with full HRQoL equals 1 QALY.^(5)^ Prognostic predictions at the time of intensive care unit (ICU) admission are often imprecise.^(6)^ Time-limited trials of intensive care may enhance prognostic assessment by identifying patients with poor expected outcomes through the evaluation of organ dysfunction over time.^(2,7-9)^ Following this "time-limited" approach, we investigated predictors of survival and QALY in cancer patients who remained alive on the fifth day after ICU admission.

## METHODS

We conducted an analysis of a prospective cohort study including adult patients with solid or hematological malignancies, admitted to two oncology referral ICUs in São Paulo, Brazil, between March 2010 and August 2011. Patients alive and still in the ICU on the fifth day were included. Ethical approval was obtained from both participating centers (CAAE: 17365713.4.1001.0065). Demographics, clinical and cancer-related variables, baseline performance status (Eastern Cooperative Oncology Group [ECOG]) and HRQoL (EQ-5D-3L), and organ dysfunction evolution over five days were collected. Survival and QALY were estimated over 24 months. Cox regression models identified prognostic factors, and patients were stratified according to the number of prognostic factors they presented. Based on this stratification, estimates for survival (Kaplan-Meier) and QALY (Zhao and Tsiatis)^(10)^ were calculated. Further details on methods available in the Supplementary Material.

## RESULTS

**Figure 1:**
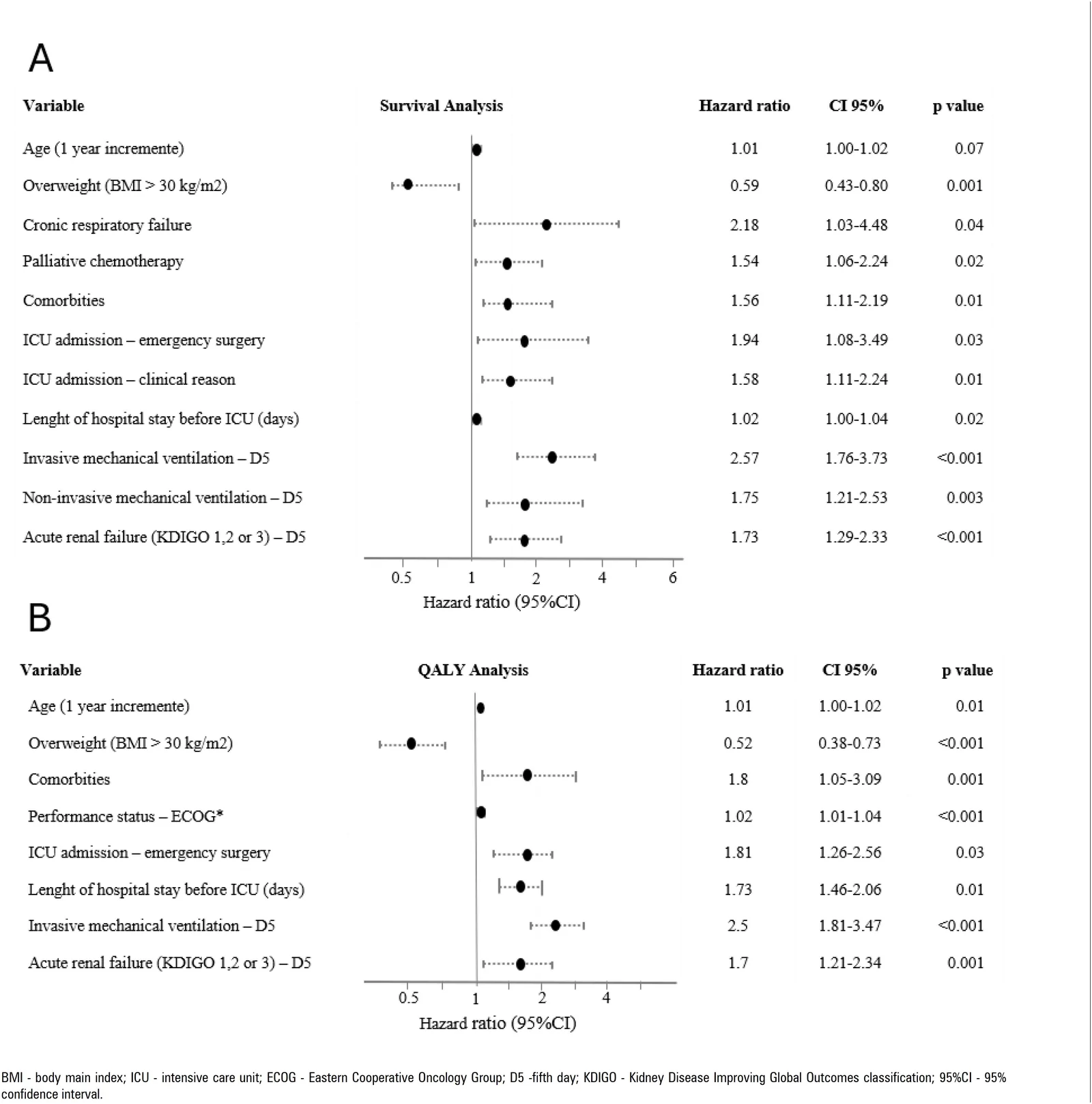
Prognosis factors identified for (A) survival and (B) QALY (Cox regression models). * ECOG (I unit increment): time-varying effect for this variable - association only during the first ten days of observation.

Of 808 screened patients enrolled in the cohort, 257 who remained in the ICU on the fifth day after admission were included (Figure 1S - Supplementary Material). The mean age was 60.7 years, 58% were male, 87% had solid tumors, and 35% had metastatic disease (Table 1S - Supplementary Material). The need for ventilatory support and vasoactive drugs, as well as changes in renal function, were assessed between the first and fifth days of ICU admission (Table 2S - Supplementary Material). Independent prognostic factors of survival included chronic respiratory failure, comorbidities, palliative chemotherapy, hospitalization for clinical reasons or after emergency surgery, longer pre-ICU hospitalization, need for non-invasive or invasive ventilatory support on the fifth day, and development of acute renal injury within the first five days. For QALY, significant prognostic factors included comorbidities, longer pre-ICU stays, baseline performance status (association only during the first ten days of observation), admission following emergency surgery, invasive ventilatory support on the fifth day, and development of acute renal injury within the first five days. Overweight was identified as a factor for improvement in both outcomes. Age was retained in the model given its established relevance in prognostic scoring systems for critically ill patients (Figure 1). Stratification by the number of prognostic factors revealed a stepwise decrease in outcomes: patients with three factors had a median survival of 38 days, and 10 days for QALY. Further details on the results are available in the supplementary material (Figure 2S - Supplementary Material).

## DISCUSSION

In this cohort study including patients with solid and hematologic malignancies who remained in the ICU for at least five days, we identified nine prognostic factors for survival, and eight prognostic factors for QALY. For both outcomes, respiratory and renal dysfunction over five days were the variables with the greatest impact on prognosis. Previous studies have identified mechanical ventilation and acute kidney injury as important prognostic markers among critically ill cancer patients, but their combined impact on long-term survival and QALY has been less explored.^(11,12)^ Despite its usual prognostic value,^(13)^ baseline performance status was not associated with survival and only had a brief association with QALY. These findings may reflect the clinical profile of patients who remained hospitalized on the fifth day, which may differ from the conditions present at hospital admission, particularly regarding acute organ dysfunction. Another aspect is that in this sample, almost half of the patients had impaired HRQoL before the critical illness, mainly in the domains of pain/discomfort and anxiety/depression, aspects that are potentially limited in the assessment of performance status. Estimates of survival and QALY stratified by the number of prognostic factors demonstrated that the accumulation of unfavorable predictors was associated with markedly worse outcomes. In our sample, the presence of three or more factors was associated with a limited survival expectancy and a markedly reduced QALY expectancy. After five days of hospitalization with unlimited treatment, identifying patients with survival and QALY estimates limited to a few weeks can help to individualize treatment.

The main limitations of this study are that the data were collected between 2011 and 2013, therefore may not capture recent advances in oncologic therapies that could modify contemporary patient prognosis; the study was conducted in specialized cancer treatment centers, which could limit the generalization of the results to non-specialized locations; the EQ-5D-3L system, used to assess HLQoL, may generate negative utility values, and it was decided to exclude patients with negative utility values from the QALY analysis to avoid methodological bias; frailty was not assessed, which could influence HLQoL by reducing physiological reserve and limiting functional recovery. Thus, although our results are not supposed to be applied in clinical practice, they strongly support further research to develop and validate prognostic models of survival and QALY among contemporary cohorts of critically ill cancer patients.

## Data Availability

The data is available upon request to the corresponding author.
