# Supplementary material for: Survival and quality-adjusted life years in critically ill cancer patients: analysis on the fifth day of intensive care unit stay
Source: Crit Care Sci. 2026 Aug 7;38:e20260443. doi: 10.62675/2965-2774.20260443 (PMC13518014; doi:10.62675/2965-2774.20260443)
Supplement: Supplementary file 1 [file 2965-2774-ccsci-38-e20260443-Suppl01.pdf]

Critical Care Science
https://doi.org/10.62675/2965-2774.20260443

SUPPLEMENTARY MATERIAL

Survival and quality-adjusted life years in critically ill cancer patients: analysis on the fifth day of intensive care unit stay

Miriam Cristine Vahl Machado[1], Antônio Carlos Pedroso-de-Lima[2], Gisela Tunes da Silva[2], Alexandre Biasi Cavalcanti[3]

METHODS

Study design and population

This study is a secondary analysis of the Normilio-Silva et al. study.(1)

The study included patients who were on their fifth day of hospitalization in the intensive care unit; patients who were discharged or who died before this day were excluded.

A complete description of the method can be found in the original study.

Variables collected

Clinical variables

Variables present on the date of intensive care unit admission (day 1)

Simplified Acute Physiology Score (SAPS) 3; type of hospitalization: surgical (elective or emergency) or non-surgical; diagnosis of nosocomial infection during intensive care unit (ICU) stay; presence of complications related to the neoplasm or its treatment (graft-versus-host disease, intracranial mass effect, hypercalcemia, mucositis, airway obstruction or compression, spinal cord compression syndrome, tumor lysis syndrome, superior vena cava syndrome, venous thromboembolic event, arterial thromboembolic event, major bleeding, liver failure, chemotherapy toxicity, adverse effect related to radiotherapy, other serious complication) - these variables were grouped as occurrence of complications; neurological status (Glasgow coma scale) and presence of delirium (Confusion Assessment Method for the ICU [CAM-ICU]); use of vasoactive drugs (noradrenaline, dopamine, dobutamine); renal function - Kidney Disease: Improving Global Outcome (KDIGO) score - creatinine criterion, need for dialysis support; assessment of performance status with the Eastern Cooperative Oncology Group (ECOG) scale and Health-related quality of life (HRQoL) - EQ-5D-3L (regarding the period prior to hospitalization).(2)

Performance status and health-related quality of life evaluation

Variables present on the 5th day in the intensive care unit

Use of vasoactive drugs (noradrenaline, dopamine, dobutamine); renal function – KDIGO (creatinine criterion), need for dialysis support; need for ventilatory support, oxygenation (PaO_2/FiO_2 ratio); neurological status, Glasgow coma scale; occurrence of complications between the first and fifth day (cardiorespiratory arrest, thromboembolic events, gastrointestinal tract bleeding, bloodstream infection, respiratory infection, surgical site infection, urinary infection, febrile neutropenia) - these variables were grouped as complications of ICU stay.

This is an open access article under the CC BY license (https://creativecommons.org/licenses/by/4.0/).

For the evolution of ventilatory support between day 1 (D1) and day 5 (D5), we chose to group invasive mechanical ventilation and non-invasive mechanical ventilation together and refer to it as "mechanical ventilation" (MV). The PaO_2/FiO_2 ratio on D5 was divided into 3 categories: less than 150, 150-200 and greater than 200.[3,4]

Quality of life adjusted survival calculation

Although Quality-Adjusted Life Years (QALY) is conventionally expressed on a yearly basis, results were rescaled and presented in days by multiplying annualized values by 365, without affecting the underlying estimates.

Statistical analysis

The analyses were carried out using R software. Numerical variables were described as mean and standard deviation or median and interquartile range (IQR) when the distribution was not normal. Categorical variables were described using absolute frequencies and percentages. The methodology used to calculate the EQ-5D-3L utility coefficients can generate negative QALY values. Since the Zhao–Tsiatis approach was not specifically developed to accommodate negative cumulative utility values.

Missing data was treated as non-informative missing. The Logrank, Gehan-Wilcoxon, and Likelihood Ratio tests (controlled for age) were used for the univariate analyses. The p-values for each level of the variables were based on the Wald test. The likelihood ratio test was used to check whether the effects of all the levels were jointly significant. The variables with a significant level of 10% were selected to be included in the multivariate Cox models. We used a variable selection based on p-value thresholds because our main goal was to assess prognosis, instead of identifying risk factors and adjusting for confounders.

Survival

The Kaplan-Meier estimator was used to analyze the data. Logrank, Gehan-Wilcoxon and likelihood ratio tests (controlled for age) were used for univariate analysis.

The independent variables were selected from previous studies showing an association with survival, e.g., SAPS 3, APACHE IV[5,6] and variables of importance in cancer patients, e.g., cancer status and extent of the disease.[1] Next, a selection method based on univariate modeling was used. Given the large number of possible predictors, the association of each variable with survival was assessed individually. The p values for each level of the variables were based on the Wald test. The likelihood ratio test was used to check whether the effects of all the levels were jointly significant.

Using a significant level of 10%, variables in the univariate model were selected to make up the initial multivariate Cox model. To build the multivariate model, the non-significant variables (considering a 5% significance level) were sequentially removed and at each stage the model was adjusted with the remaining variables. The final multivariate model was built by combining the categories whose effects were equal (p values > 0.05): in the BMI category underweight and normal weight individuals have the same survival (p = 0.506); in the renal function category the same effect is observed for chronic renal failure and normal renal function (p = 0.896), as well as renal dysfunction stages 1, 2 or 3 having the same effect on survival (p = 0.813); patients undergoing curative chemotherapy have the same survival as those not undergoing chemotherapy (p= 0.471). The assumption that the proportional rates had not been violated in the multivariate analysis was verified.

Following this, the prognostic factors identified in the multivariate analysis were dichotomized according to risk (low or high), and the distribution of patients according to the number of prognostic factors presented was assessed. Patients were grouped according to whether they had 0 - 1 prognostic factor, 2 prognostic factors, 3 prognostic factors, 4 prognostic factors, 5 or more prognostic factors. Kaplan-Meier curves were generated from these categories to estimate survival according to the stratification of patients by the number of prognostic factors presented.

Quality of life adjusted survival – QALY

The survival curves for QALY were estimated using the method described by Zhao-Tsiatis[7] as an adaptation. This method was chosen because of the information censorship induced by combining survival with quality of life, resulting in QALY. Traditional methods, such as Kaplan-Meier, presuppose non-informative censoring, making them unsuie 3Se in this case.

The EQ-5D-3L system, the methodology used to construct QALY, can generate negative utility values. The Zhao–Tsiatis method was used as an alternative approach for QALY survival curve analysis; however, it was not originally developed to handle negative cumulative utility values. Inclusion of negative QALYs may lead to unstable estimates and challenging interpretation of the integrated survival–utility function; therefore, these cases

were excluded from the analysis. Hence, of the 257 observations, 29 patients were excluded.

The p values for each level of the variables were based on the Wald test.

The variables identified in the univariate model with a significant level of 10% were selected to make up the initial multivariate Cox model. To obtain a more parsimonious model, the effects of the levels of the variables in the previous model were checked to see whether they could be combined. It was observed that for BMI, low weight has a similar effect to normal weight ($p = 0.823$); type of admission after elective surgery has the same effect as clinical admission ($p = 0.154$); the levels of ventilatory support evolution between D1 - D5 "Without MV → Without MV" and "With MV → Without MV" have the same effect (p = 0. 530), evolution of renal function D1-D5 without dysfunction and (previous) chronic renal failure had the same effect ($p = 0.150$) and levels of renal dysfunction between D1 - D5 with stages 1,2 or 3 had effects that were not statistically different ($p = 0.1789$ - likelihood ratio test).

For the multivariate Cox model, the assumptions that the proportional rates had not been violated were verified. A test based on Schoenfeld residuals was initially used, as well as additional Kolmogorov-Smirnov and Cramér-von Mises proportionality tests. It was identified that the ECOG variable violated the proportionality assumption. The model was then adjusted, considering a time-varying effect for this variable (positive effect between 0 and 10 days; no effect of this variable on QALY after the tenth day).

The prognostic factors identified in the multivariate analysis were dichotomized according to risk (low or high). Subsequently, the distribution of patients according to the number of prognostic factors presented was assessed. Patients were grouped according to whether they had 0 or 1 prognostic factor, 2 prognostic factors, 3 prognostic factors, 4 prognostic factors, 5 or more prognostic factors. Curves were generated using the Zhao-Tsiatis method to estimate QALY based on the stratification of patients according to the number of prognostic factors.

RESULTS

Characteristics of the patients

Between March 2011 and August 2012, 808 patients were admitted to ICUs and assessed for eligibility, and 792 patients were included in the study. Of these, 469 were discharged and 65 died before the fifth day of hospitalization, resulting in 257 patients who remained in the ICU on the fifth day and were included in the analysis (202 at *Instituto do Câncer do Estado de São Paulo* [ICESP] and 55 at the *Fundação Pio XII*) (Figure 1S). Follow-up ended in September 2013. The baseline characteristics are shown in table 1S. The evolution of organ dysfunctions between the first (D1) and fifth day (D5) is shown in table 2S.

Quality-Adjusted Life Years

The stratification of patients based on the number of risk factors presented is shown in figure 2S.

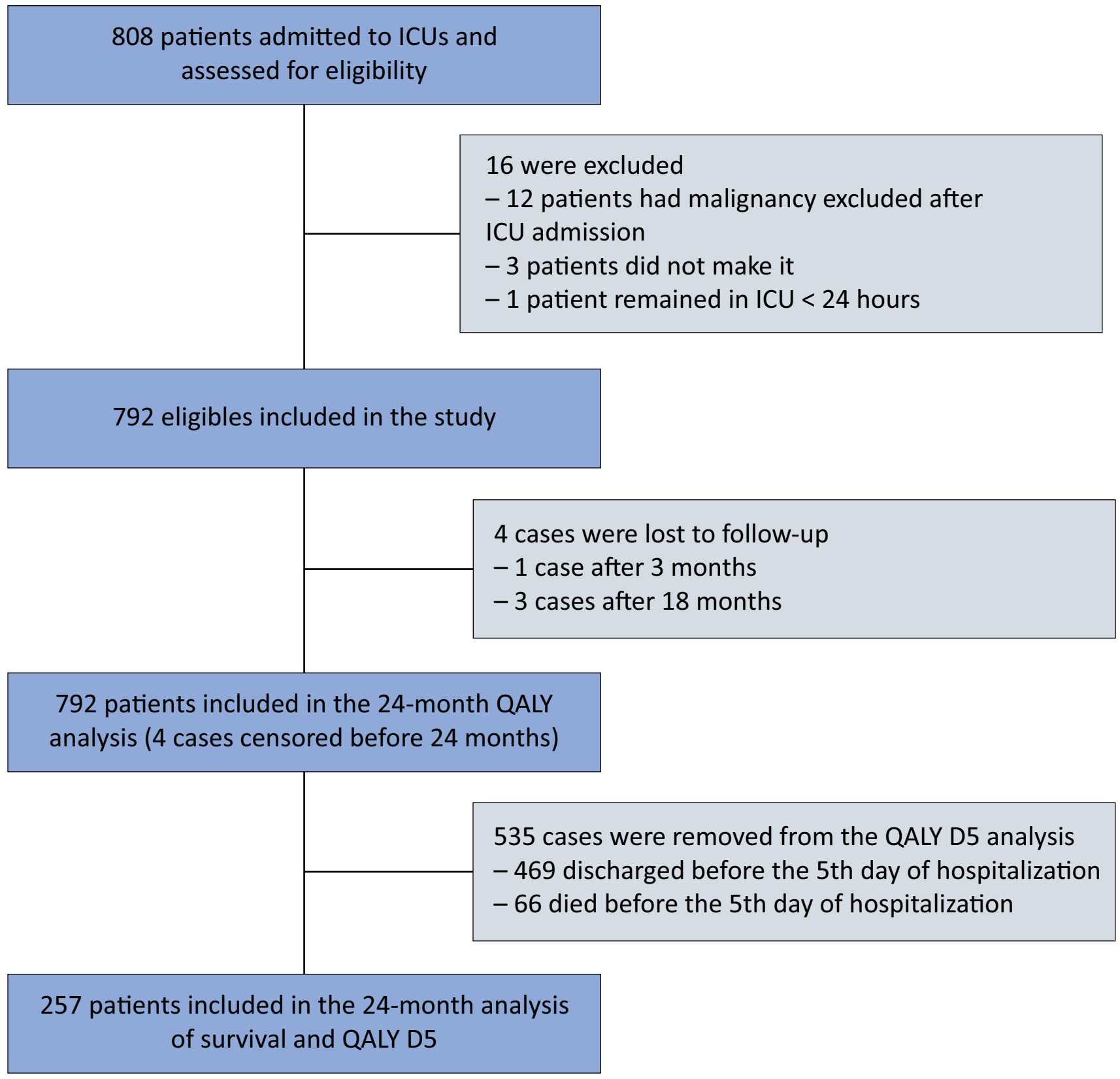

ICU - intensive care unit; QALY - Quality-Adjusted Life Years; D5 - fifth day.

Figure 1S - Included patients.

Table 1S - Baseline characteristics

Characteristics	
Demographic/anthropometric data	
Age (years)	61.14 ± 14.88
Sex Male	58
Body mass index (Kg/m^2)	24.2 ± 5.4
SAPS 3	55.1 ± 13.83
Comorbidities	
Chronic renal failure	11 (4)
Chronic respiratory failure	10 (4)
Cirrhosis	7 (3)
Congestive heart failure (NYHA III-IV)	13 (5)
Diabetes	36 (14)
Alcoholism	30 (12)
Primary location	
Digestive tract	87 (34)
Genital system	31 (12)
Hematological	29 (11)
Urinary tract	23 (9)
Respiratory system	21 (8)
Breast	18 (7)
Oral cavity and pharynx	17 (7)
Brain and central nervous system	15 (6)
Other/not determined	16 (6)
Neoplasm status	
Active, relapse/progression	125 (49)
Active recent diagnosis	118 (46)
Controlled/remission	14 (5)
Extension of the disease	
Distant metastasis	90 (35)
Locally advanced	83 (32)
Limited	77 (30)
Hematologic (leukemias)	7 (3)
Chemotherapy/Radiotherapy	
Chemotherapy	124 (48)
Curative/adjuvant intent	82 (32)
Palliative intent	42 (16)
Radiotherapy	76 (30)
Curative/adjuvant	60 (23)
Palliative	16 (6)
Type of admission	
Clinical admissions	167 (65)
Elective surgeries	72 (28)
Emergency surgery	18 (7)
Clinical reasons for hospitalization	
Sepsis	42 (25)
Pneumonia	20 (12)
ARDS	15 (9)
Other respiratory complications	14 (8)
Miscellaneous reasons	75 (45)
Hospital days before ICU	
< 1 day	74 (29)
1 - 7 days	45 (18)
> 7 days	138 (54)
ECOG	
0 - fully active	79 (31)
1 - restricted for physically strenuous activity	72 (28)
2 - ambulatory and capable of all self-care but unable to carry out work activities	45 (18)
3 - capable of only limited self-care, confined to bed/ chair > 50% time	41 (16)
4 - completely disabled, cannot carry on any self-care, totally confined to bed/chair	20 (8)
EQ-5D-3L utility coefficients (before critical illness)	
< 0,4	119 (46)
0,4 - 0,6	20 (8)
0,6 - 0,8	68 (26)
0,8 - 1,0	50 (19)
Distribution of domains - EQ-5D-3L	
Mobility	
1 - No problem	129 (50)
2 - Some problems	96 (37)
3 - Extreme problems	32 (13)
Self-care	
1 - No problem	154 (60)
2 - Some problems	71 (28)
3 - Extreme problems	32 (12)
Usual activities	
1 - No problem	105 (41)
2 - Some problems	96 (37)
3 - Extreme problems	56 (22)
Pain/discomfort	
1 - No problem	75 (29)
2 - Some problems	118 (46)
3 - Extreme problems	64 (25)
Anxiety/depression	
1 - No problem	84 (33)
2 - Some problems	111 (43)
3 - Extreme problems	62 (24)

SAPS - Simplified Acute Physiology Score; NYHA - New York Heart Association; ARDS - acute respiratory distress syndrome; ICU - intensive care unit; ECOG - Eastern Cooperative Oncology Group. Results expressed as mean ± standard deviation; n or n (%).

Table 2S - Organ dysfunctions and complications in the period between the first and fifth day

Characteristics	n (%)
Ventilatory support	N = 257
No ventilatory support required	102 (40)
MV on D1 - No MV on D5	48 (19)
MV on D1 - MV on D5	35 (13)
No MV in D1 - MV in D5	61 (24)
No assessment	11 (4)
Oxygenation - PaO_2/FiO_2 ratio	N = 257
$PaO_2/FiO_2 < 150$	75 (29.2)
PaO_2/FiO_2 150 - 200	31 (12.1)
$PaO_2/FiO_2 > 200$	150 (58.4)
No assessment	1 (0.3)
Vasopressor	N = 257
No use required	143 (55)
Decreasing dose	51 (20)
Increasing dose	59 (23)
No assessment	4 (2)
Renal function - KDIGO	N = 257
No renal dysfunction	150 (58.3)
Grade 1 dysfunction	29 (11.3)
Grade 2 dysfunction	30 (12)
Grade 3 dysfunction	34 (13.2)
Chronic renal failure	11 (4.5)
No assessment	3 (0.2)
Occurrence of complications	N = 257
No	168 (65)
Yes	89 (35)
Occurrence of infections	N = 257
Yes	65 (25)
No	192 (75)
Respiratory system	27 (10.5)
Bloodstream	15 (6)
Surgical site	7 (3)
Urinary tract	6 (2.5)
Febrile neutropenia	5 (2)
Other sites	5 (2)

MV - mechanical ventilation; D1 - first day; D5 - fifth day; KDIGO - Kidney Disease Improving Global Outcomes classification;

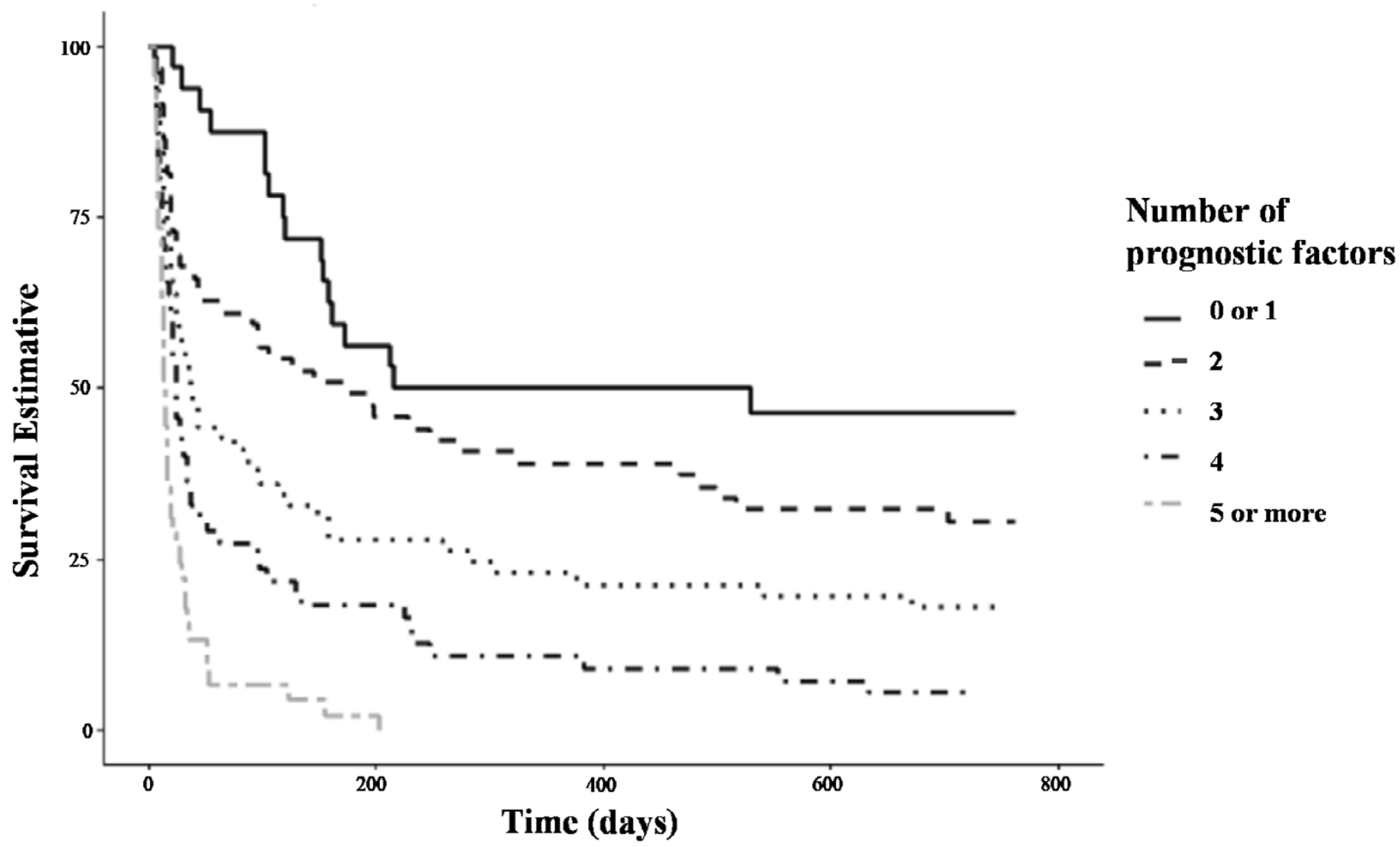

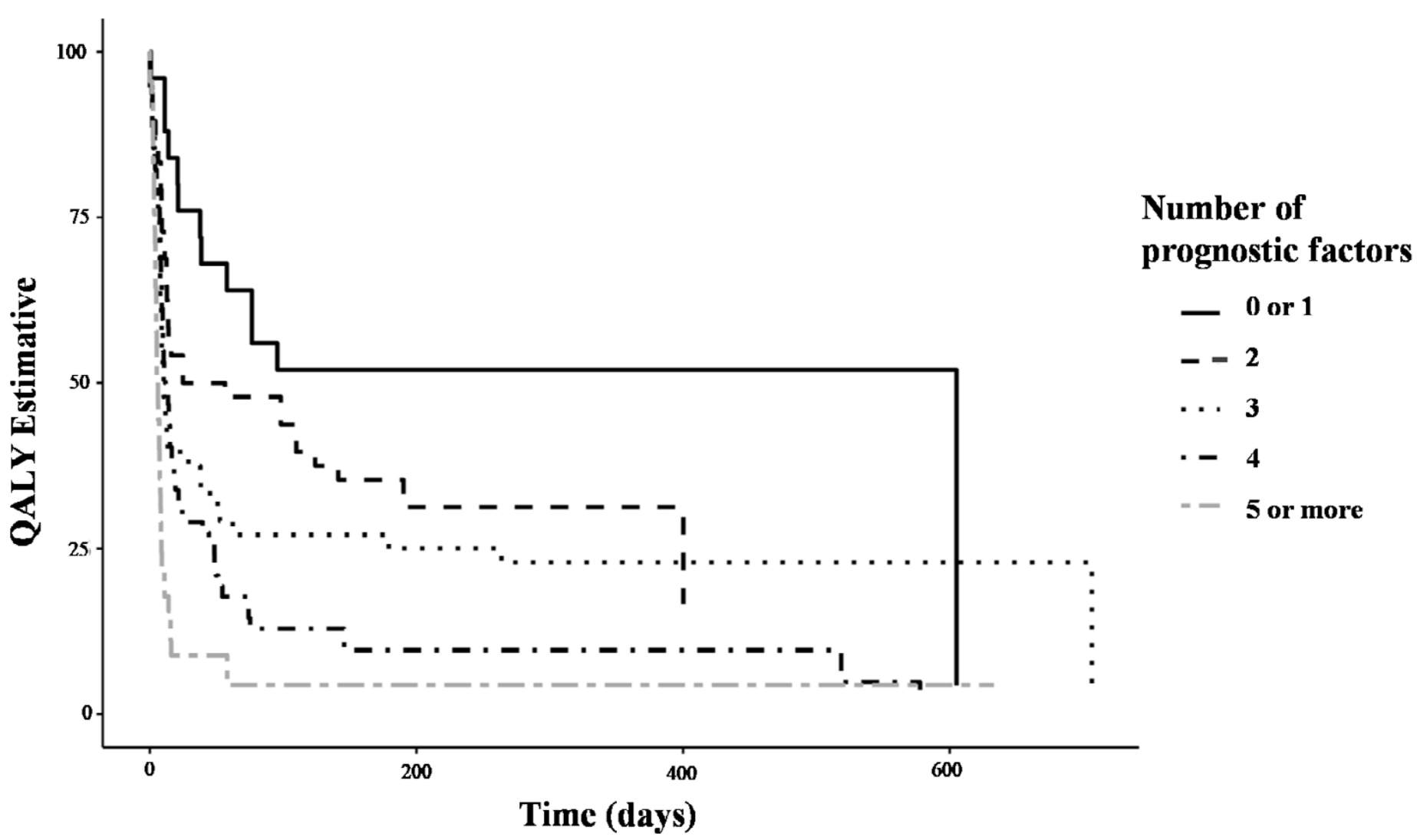

Prognostic Factors Number	Survival* N (%)	Survival (days) median (IQR; 95% IC)	QALY ** N (%)	QALY (days) median (IQR)
0 or 1	32 (13)	373 (120 - #; 158 - # #)	25 (11)	605 (38 - #)
2	59 (23)	175 (20 - #; 66 - 500)	48 (21)	25 (9 – 400)
3	61 (24)	38 (17 – 282; 25 - 113)	48 (21)	10 (6 – 149)
4	55 (22)	24 (12 – 98; 20 - 37)	62 (27)	11 (7 – 48)
5 or more	45 (18)	15 (9 – 28; 12 - 19)	45 (20)	6 (4 – 9)

Of the total of 257 patients, 04 have no information on the mechanical ventilation variable, and 01 has no information on chemotherapy.
**A total of 29 patients with negative QALY were excluded. # non-estimable quartile. # # non-estimable limit.
IQR: interquartile range

Figure 2S - Estimates of survival (Kaplan-Meier) and QALY (Zhao-Tsiatis) according to group stratification.
* Of the total of 257 patients, 4 have no information on the mechanical ventilation variable, and 1 has no information on chemotherapy; ** a total of 29 patients with negative QALY were excluded; # non-estimable quartile; ## non-estimable limit.

REFERENCES

1. Normilio-Silva K, de Figueiredo AC, Pedroso-de-Lima AC, Tunes-da-Silva G, Nunes da Silva A, Delgado Dias Levites A, et al. Long-term survival, quality of life, and quality-adjusted survival in critically ill patients with cancer. Crit Care Med. 2016;44(7):1327-37.
2. Pickard AS, Wilke CT, Lin HW, Lloyd A. Health utilities using the EQ-5D in studies of cancer. PharmacoEconomics. 2007;25(5):365-84.
3. Villar J, Pérez-Méndez L, Kacmarek RM. Current definitions of acute lung injury and the acute respiratory distress syndrome do not reflect their true severity and outcome. Intensive Care Med. 1999;25(9):930-5.
4. Rouby JJ, Puybasset L, Cluzel P, Richecoeur J, Lu Q, Grenier P; CT Scan ARDS Study Group. Regional distribution of gas and tissue in acute respiratory distress syndrome. II. Physiological correlations and definition of an ARDS Severity Score. Intensive Care Med. 2000;26(8):1046-56.
5. Zimmerman JE, Kramer AA, McNair DS, Malila FM. Acute Physiology and Chronic Health Evaluation (APACHE) IV: hospital mortality assessment for today's critically ill patients. Crit Care Med. 2006;34(5):1297-310.
6. Moreno RP, Metnitz PG, Almeida E, Jordan B, Bauer P, Campos RA, et al; SAPS 3 Investigators. SAPS 3 - From evaluation of the patient to evaluation of the intensive care unit. Part 2: Development of a prognostic model for hospital mortality at ICU admission. Intensive Care Med. 2005;31(10):1345-55.
7. Cesar RC. Estimação e comparação de curvas de sobrevivência sob censura informativa [tese]. São Paulo: Instituto de Matemática, Estatística e Ciência da Computação, Universidade de São Paulo; 2013.
